# Shape-Morphing of an Artificial Protein Cage with Unusual Geometry Induced by a Single Amino Acid Change

**DOI:** 10.1021/acsnanoscienceau.2c00019

**Published:** 2022-05-09

**Authors:** Mohit Sharma, Artur P. Biela, Agnieszka Kowalczyk, Kinga Borzęcka-Solarz, Bernard M. A. G. Piette, Szymon Gaweł, Joshua Bishop, Philipp Kukura, Justin L. P. Benesch, Motonori Imamura, Simon Scheuring, Jonathan G. Heddle

**Affiliations:** † Malopolska Center of Biotechnology, Jagiellonian University, Gronostajowa 7A, Kraków 30-387, Poland; ‡ School of Molecular Medicine, Medical University of Warsaw, Warsaw 02-091, Poland; § Faculty of Mathematics and Computer Science, Jagiellonian University, Kraków 30-348, Poland; ∥ Department of Mathematical Sciences, University of Durham, Durham DH1 3LE, U.K.; ⊥ Department of Chemistry, University of Oxford, Oxford OX1 3TA, U.K.; # Kavli Institute for Nanoscience Discovery, University of Oxford, Oxford OX1 3QU, U.K.; ∇ Department of Anesthesiology, Weill Cornell Medicine, New York City, New York 10065, United States; ○ Department of Physiology and Biophysics, Weill Cornell Medicine, New York City, New York 10065, United States

**Keywords:** artificial protein cage, TRAP, point mutation, symmetry breaking, bionanoscience, programmable matter

## Abstract

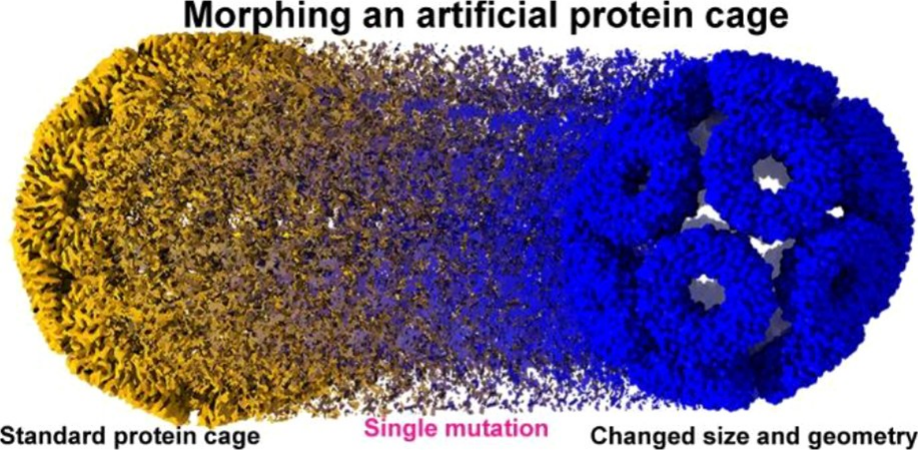

Artificial protein cages are constructed from multiple protein subunits. The interaction between the subunits, notably the angle formed between them, controls the geometry of the resulting cage. Here, using the artificial protein cage, “TRAP-cage”, we show that a simple alteration in the position of a single amino acid responsible for Au(I)-mediated subunit–subunit interactions in the constituent ring-shaped building blocks results in a more acute dihedral angle between them. In turn, this causes a dramatic shift in the structure from a 24-ring cage with an octahedral symmetry to a 20-ring cage with a C2 symmetry. This symmetry change is accompanied by a decrease in the number of Au(I)-mediated bonds between cysteines and a concomitant change in biophysical properties of the cage.

## Introduction

**Figure 1:**
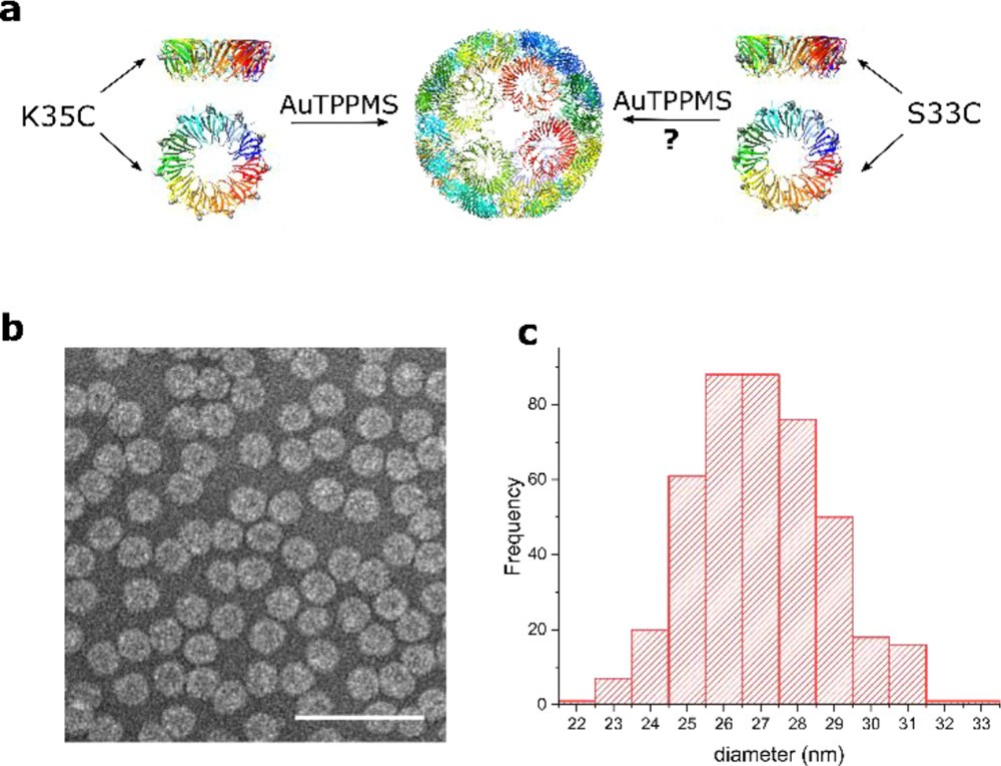
TRAP^S33C-Au^-cage design and TEM analysis: (a) scheme of TRAP^K35C-Au^-cage assembly (left) with putative scheme of the same reaction for TRAP^S33C-Au^-cage. (b) TEM image of the TRAP^S33C-Au^-cage (scale bar 100 nm). (c) Distribution of apparent diameter of TRAP^S33C-Au^-cages measured on TEM images showed that the most abundant species are of approximately 26–28 nm in diameter.

Protein cages are hollow, nanoscale protein structures. They are widespread in nature, with naturally occurring varieties such as viruses, serving as inspiration for recent advances, allowing the design and production of artificial cages.^1−3^ The most successful examples are computationally designed protein cages that recapitulate native-like protein–protein interfaces between protein building blocks of appropriate symmetry, which otherwise do not form cages in the native state.^4−7^ A challenge in this approach lies in designing the multiple amino-acid interactions found in protein–protein interfaces. This can be overcome with alternative methodologies, whereby such interactions are replaced with fewer, simple connections such as coordinate bonds with metals. A small number of such cages have been reported including Zn(II)- and/or Fe(III)-mediated cage assembly.^8^ We previously reported the use of trp RNA-binding attenuation protein (TRAP) to build an artificial protein cage (“TRAP-cage”). TRAP is a bacterial protein typically constructed of eleven identical monomers with the resulting complex being an approximately 90 kDa ring. TRAP has been well-characterized structurally and biochemically^9−14^ and has been developed as a biotechnological tool, including for the construction of artificial structures such as cages and rings.^15−21^ Recently, TRAP-cage has been further modified and shown capable of delivering cargoes to the interior of human cells.^21^ TRAP-cage is constructed from 24 copies of TRAP containing a single cysteine at amino acid 35 in place of the native lysine (TRAP^K35C^, Figure 1a, left).^16−18^ The TRAP-cage is held together solely by bonds between the cysteines involving a bridging Au(I). One outcome of this unique structure is that cage assembly only occurs when Au(I) is present (assembly is triggerable). Disassembly is also triggerable and programmable: thiol-bearing compounds able to carry out exchange reactions with the Au(I) such as DTT cause rapid disassembly of the cage which is otherwise highly stable^18^ while Au(I) itself has the potential to be replaced with other “linkers” with predictable cleavage properties (programmable disassembly). This was recently demonstrated in work where molecular cross-linkers dithiobismaleimidoethane and bismaleimidohexane, which have contrasting cleavage responses to reducing conditions, were used to produce programmable TRAP-cages. These were shown capable of encapsulating stable functional protein cargoes, which were freed upon exposure to a specific disassembly trigger.^22^ Stable protein cages carrying cargoes that can be released upon a trigger of choice including reducing conditions have clear potential applications in the delivery of macromolecular therapeutics to cells (given the reducing nature of the cellular cytoplasm).

The introduced cysteine residue at position 35 lies at a prominent position on the outer rim of the TRAP ring and is repeated 11 times as each ring is made from 11 identical TRAP monomers. Its positioning is near-optimal, resulting in a dihedral angle between rings of 135.3° up to 137.3°,^18,23^ close to the optimal of 136.31° seen between the pentagonal faces of a pentagonal icositetrahedron (PI), the Catalan solid (specifically, the TRAP-cage can be viewed as approximating the shape achieved if a TRAP ring is inscribed within each pentagonal face of the PI).

The previously studied TRAP^K35C-Au^-cage, consisting of 24 TRAP rings, is likely to represent the energetically most favored arrangement for a cage made from multiple copies of a hendecamer with optimal angles as described above. Given this, we asked the question how might the overall shape and geometry of TRAP-cages employing cysteines at different positions change their shape and geometry to accommodate a less energetically favored set of interactions? This could be tested by keeping all other parameters constant (i.e., using the same protein and gold cross-linker) and changing only the position of the cross-linked cysteine. Our original rationale for choosing the alternative positions for cysteines was that they should be prominent, surface-exposed residues that lie on the surface of the TRAP monomer such that in the assembled ring, they may be able to form lateral ring-ring connections necessary for assembly into cages. The fact that TRAP is a small protein (74 residues) with significant buried protein–protein interfaces restricts the number of potential locations for the cross-linked cysteine considerably. We previously identified residues D15 and S33 as the only favorable potential candidates and showed that D15 is apparently unable to support Au(I)-mediated cage formation while TRAP bearing mutation S33C does appear to result in cage formation as assessed by native PAGE and negative stain transmission electron microscopy (TEM) analysis.^18^ Residue S33, like K35, lies on the outer “rim” of the TRAP ring which can also be thought of as a highly truncated cone, wherein, in the assembled cage, the narrow end of the cone points to the interior lumen. Structural models show that S33 is located further toward this narrow end of the cone compared to K35 (Figure 1a). This implies that the angle between rings and therefore the geometry of the resulting cages should differ from that formed with TRAP bearing the K35C mutation. How cage assembly with the S33C mutant occurs and the resulting structure is currently unknown and is addressed in this work. We show that as a result of the altered interaction between the rings, the geometry of the cage is indeed changed, leading to the formation of TRAP-cages containing fewer rings and with fewer bonds between rings.

This work gives us further insight into the parameters affecting TRAP-cage assembly and further insight into the applicability of mathematical models in predicting likely cage structures. We expect the data to be useful in further bespoke design of TRAP-cages and other artificial protein cages.

## Results

### TEM Analysis

We purified modified TRAP rings and assembled them into TRAP-cages in the presence of Au(I) using methods similar to those previously described^18^ (see Methods section). The resulting cage, named TRAP^S33C-Au^-cage, was initially confirmed using negative stain TEM. In line with previous results,^18^ this showed clear circular structures consistent with a close-to-spherical cage morphology (Figure 1b). Apparent diameter ranged from 23 to 31 nm (Figure 1c) with the majority falling within a narrow range (26–28 nm). This compares to 22 nm as determined by dynamic light scattering for the TRAP^K35C-Au^-cage.^18^ As negative stain TEM gives only 2D information and is not of sufficient resolution to enable quantitation of the number of TRAP rings in the structure, we next carried further experiments to gain a more accurate measurement of size and further insight into structural details.

### Initial Structure Prediction Using High-Speed AFM and Mass Photometry

**Figure 2:**
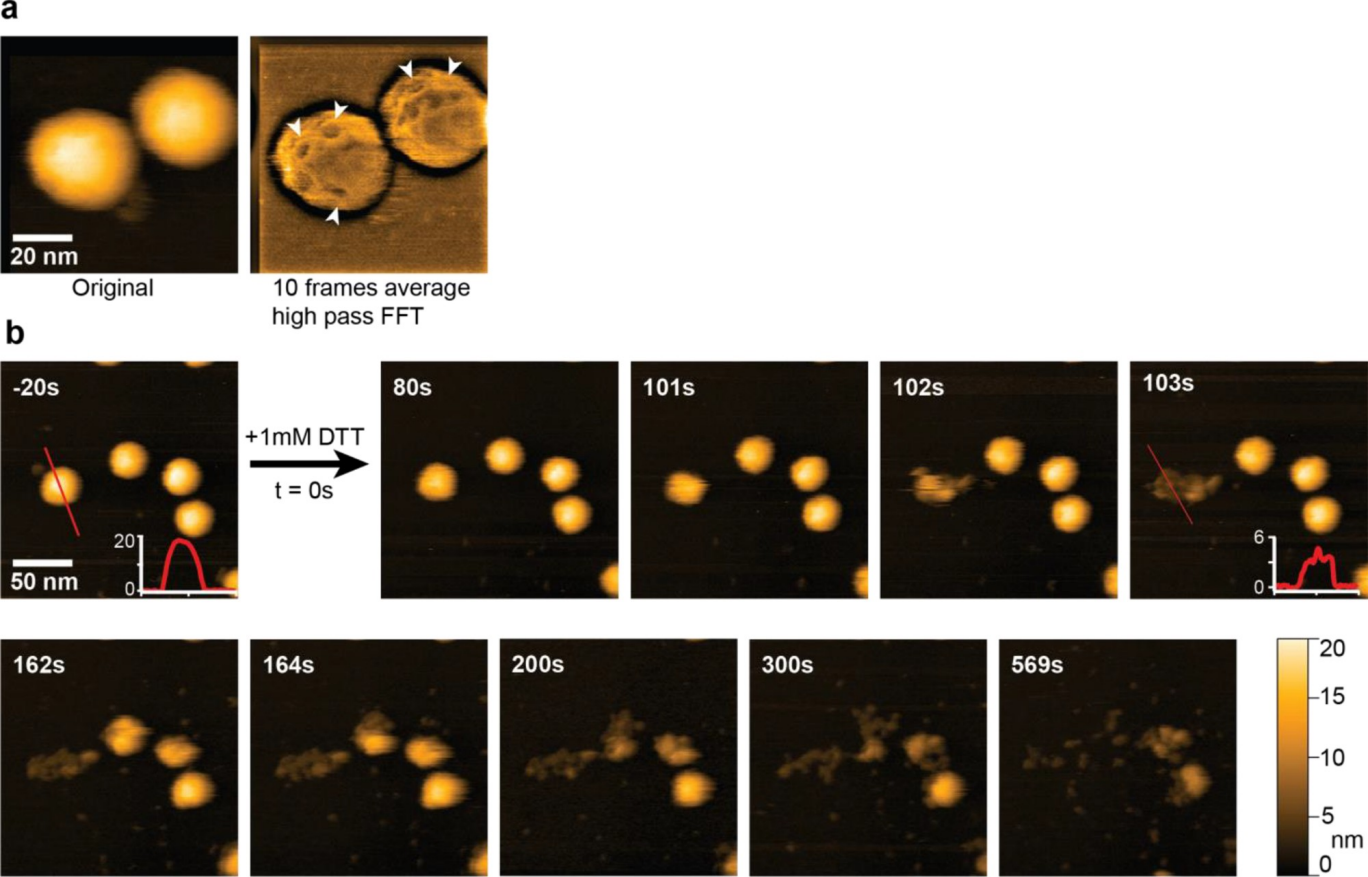
High speed atomic force microscopy of (a) intact TRAP^S33C-Au^-cages of original image and processed with high pass FFT filter and 10 frames averaging, showing the spherical structure of TRAP^S33C-Au^-cages with ring structures on the cages’ surface (indicated by arrows). Also, (b) showing the time-dependent disassembly of the cages upon 1 mM DTT addition at *t* = 0. Height profiles, shown in the insets (80 nm × 20 nm or × 6 nm). AFM images were taken at 1 frame per second, 80 nm × 80 nm or 200 nm × 200 nm, 200 pixel ×200 pixel. Time stamp in the upper left corner of each AFM image indicates time after DTT addition. Scale bar is 20 nm in a and 50 nm in b.

To gain more information on the TRAP^S33C-Au^-cage structure, we carried out high-speed AFM (HS-AFM). This technique also allowed us to probe the response of the cage to reducing agent. We investigated cage in the presence and absence of 10 mM DTT (Movie S1 and Movie S2, respectively). These analyses showed us that indeed TRAP^S33C-Au^-cages are approximately spherical and constructed from TRAP rings, which are clearly visible after high pass filtering and frames averaging (Figure 2a, Movie S2). Addition of 10 mM DTT resulted in the disassembly of the cages within 120 s (Figure 2b, Movie S1). This suggested that, analogous to TRAP^K35C-Au^-cages, these cages were held together by Au(I) ions bridging opposing cysteines, whereby the cage is disassembled by the gold-etching effects of DTT.^18^

To further understand the structure of the TRAP^S33C-Au^-cage, we conducted mass photometry analyses of the assembled cage to try to determine its molecular mass and hence the number of constituent TRAP rings. The results (Figure S1) gave an average mass of 1.8 MDa. This is considerably below the 2.2 MDa measured for the TRAP^K35C^-cage, which consists of 24 rings.^18^ Assuming an empty cage in both cases, then, simplistically, this would imply a cage of 20 rings for the TRAP^S33C^Au-cage. However, this is complicated by the possible presence of cargo in the cage and the broad peak observed at each timepoint implies a range of masses (possibly consistent with the variation in the amount of cargo as well as incompletely formed or partially disrupted cages).

### TRAP^S33C^-cage Stability

**Figure 3:**
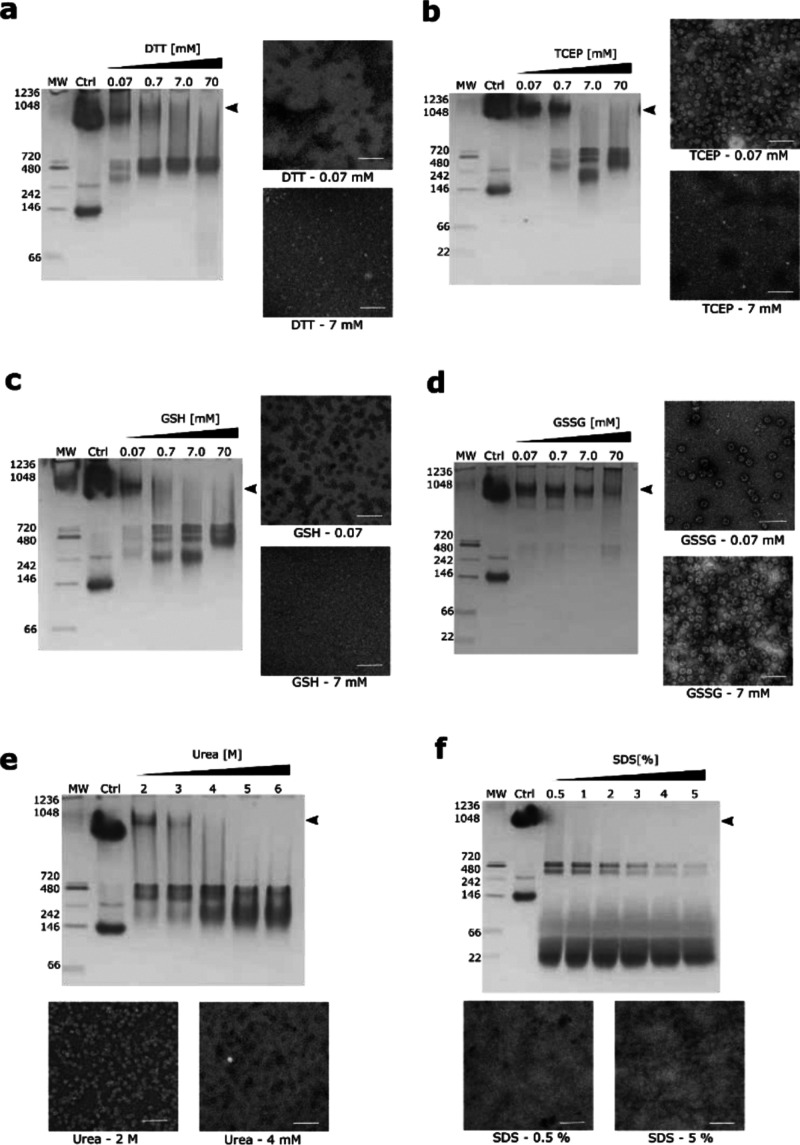
Chemical stability of TRAP^S33C-Au^-cage. Native PAGE shows the preservation of structural integrity at the different conc. (mM, M and percentage) in the presence of different reducing agents. (a) Stability in the presence of DTT; no visible loss of structure was seen up to 0.7 mM conc. (b) Stability in the presence of TCEP; no visible loss of structure is seen before 0.7 mM conc. (c) Stability in the presence of GSH; no visible loss of structure is seen up to 0.7 mM. (d) Stability in the presence of GSSG; no visible loss of structure is seen up to 70 mM. (e) Effect of UREA; TRAP-cage is stable at 2 M. (f) Effect of SDS; no resistance of the TRAP^S33C-Au^-cage is seen; scale bar 100 nm, arrowhead indicates the assembled cage.

The position of the S33C mutation is closer to the narrow face of the TRAP ring, which points to the cage interior in the known TRAP-cage structure.^18^ Assuming a similar cage structure formed by the TRAP^S33C^-cage would imply that the dihedral angle between rings will become more acute in order to satisfy the bond length requirements of binding Au(I). In turn, this could lead to changes in other interactions, which may affect the stability of the resulting cage. To investigate effects on stability, we probed cage integrity under a number of conditions (for details, see Methods). Cage disassembly was first probed in reducing conditions using a combinationof native PAGE, HS-AFM and mass photometry. The addition of DTT (Figure 3a, Figure S1, Movie S1) and TCEP (Figure 3b) disassembled the cage completely at 70 and 7 mM, respectively. The reduced form of glutathione (GSH) was also able to disrupt the integrity of the cage (Figure 3c). In contrast, the oxidized form of glutathione had no effect on cage integrity within tested conditions (Figure 3d). Significant stability against chaotropic agents was observed, with transition point between 2 and 3 M urea (Figure 3e). Unlike the TRAP^K35C-Au^-cage, the TRAP^S33C-Au^-cage appeared to be significantly more sensitive to detergents such as SDS, showing complete disassembly at only 0.5% of the agent (Figure 3f).

Thermal stability of the TRAP^S33C-Au^-cage was also tested along with resistance against a wide range of pHs (Figure S2). Native-PAGE and TEM analysis (Figure S2a,b) showed that the cage was stable at pH 6–8 with some proportion remaining as intact cages up to pH 10. For thermal stability, the measurement of the inflection point on a thermal denaturation curve (Figure S2c, see Methods) was found to be 76.8 °C. This is consistent with results after applying heated cage samples on native PAGE gels, which showed that the incubation of the TRAP^S33C-Au^-cage for 30 or 90 min resulted in the loss of structure for the majority of cages at 65–85 °C. Approximately, 50% of the cages appear disassembled after 5 min incubation at 95 °C (Figure S2d).

Overall, these results suggest that the TRAP^S33C-Au^-cage forms a stable cage, which is sensitive to reducing agents, consistent with the results seen for the TRAP^K35C^-based cage. Differences to the TRAP^K35C-Au^-based cage lie in the extent of stability, with TRAP^S33C-Au^-cage being generally less stable and particularly in the response to SDS, where the TRAP^S33C-Au^-cage appears to be particularly sensitive.

### Cryo-EM

The overall structure of the TRAP^S33C-Au^-cage was determined using cryo-EM (Figure S3, Table S1). Analysis of the reconstructed map showed that the TRAP^S33C-Au^-cage is composed of twenty TRAP^S33C^ rings, in agreement with the mass photometry measurements. As a result, the overall symmetry of the new cage structure is significantly lower than that determined for the TRAP^K35C-Au^-cage, changing from octahedral to C2. This means that the cage itself is no longer spherical, but slightly elongated in the C2 axis direction. This is clearly observable in radius-dependent coloring of the reconstructed density (Figure S4a–d).

Local resolution estimations within the reconstructed density (Figure S4e–h) clearly show a region of lower resolution located at one end of the particle along the C2 axis. This phenomenon could be due to extended flexibility of the cage in this particular direction, or a lower occupancy of this region meaning that some analyzed cages were missing ring(s) in this position.

**Figure 4:**
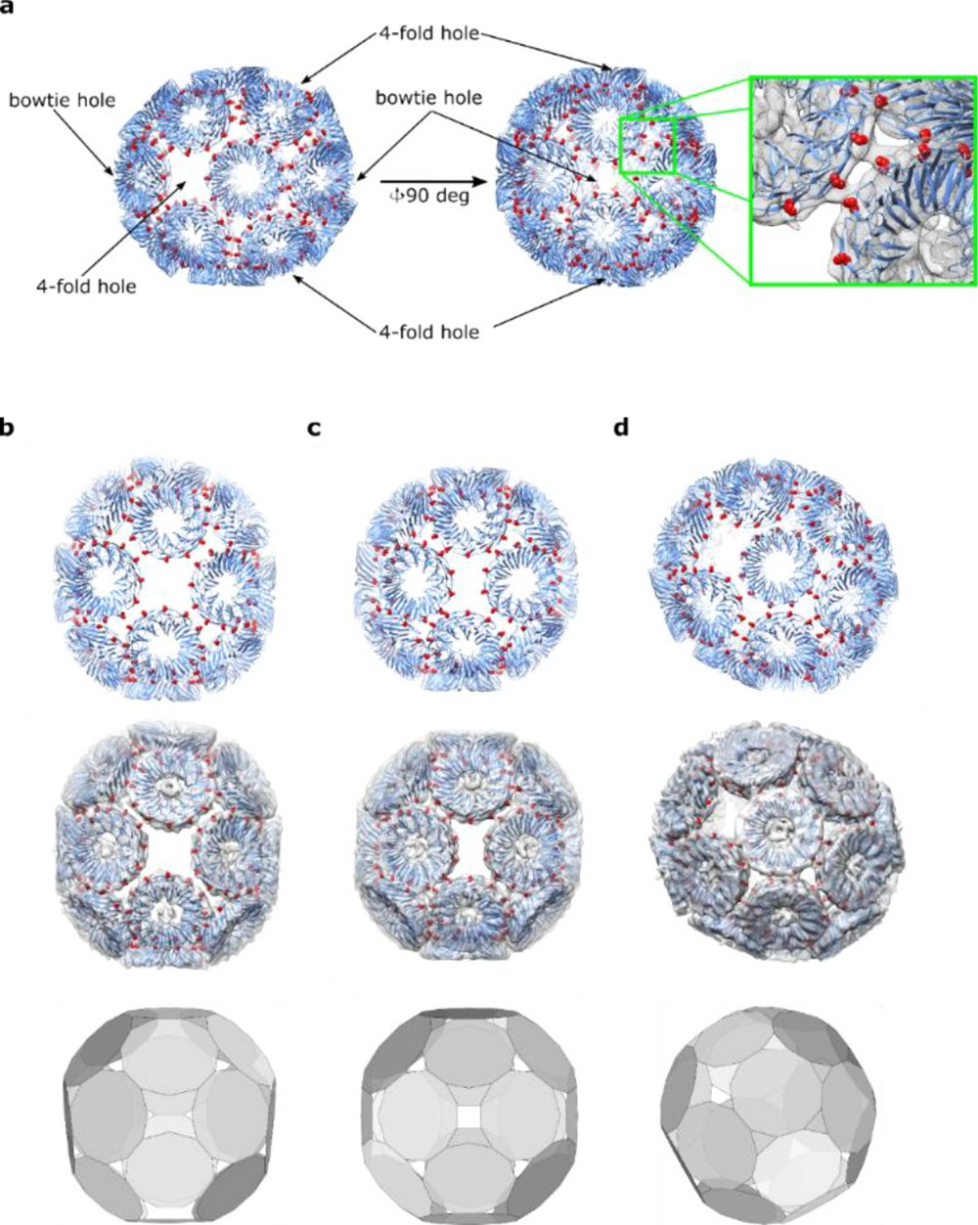
Analysis of an overall geometry of the TRAP^S33C-au^-cage. (a) Two orthogonal views of a pseudo atomic model of the TRAP^S33C-Au^-cage with enlarged section showing the position of the Cys residues in two adjacent rings inside reconstructed density (protein part shown as a blue ribbon, Cys residues as red spheres, density in gray mesh. (b) View centered on the four-fold hole. (c) View centered on bowtie hole; (d) perspective view (in b–d pseudo atomic model, pseudo atomic model in reconstructed density, and mathematically simulated model are displayed in the top, middle, and bottom panels, respectively).

Closer examination of the assembled cage structure revealed that the 20 TRAP^S33C^ rings are connected with bridging densities reminiscent of those seen in the TRAP^K35C-Au^-cage^18^ (Figure 4a). This gives confidence that despite the poorer resolution, in this case, the connections between adjacent rings are the same gold staples as seen in the previous TRAP-cage, with Au(I) ions acting like bridges between two opposing Cys residues. These bridges span a distance of >5 Å between sulfurs – considerably too long to be direct disulfide bonds (Figure S5). Ninety-six such bridges could form between the twenty rings of the cage. However, we saw occasional examples of incomplete, or even complete lack of, bridging densities, implying less than the maximal number of bridges may be formed (Figure S5). This can be a result of limited flexibility of the protein rings, making them incapable of completely satisfying all possible connections predicted by the mathematical model. Additionally, lower than 100% occupancy of the gold positions in the cage structure may be directly linked to the flexibility of the protein rings mentioned above.

We noted two types of large holes in the cage. One resembles the regular four-fold hole (Figure 4b) that can be found in the TRAP^K35C-Au^-cage (of which there are four in the new cage, each having four unsaturated Cys residues). The second resembles a bowtie shape (Figure 4c), and there are two of them, each having six unsaturated Cys residues. Bearing in mind that the full cage is composed of 220 TRAP monomers, there are 220 Cys residues, but only a maximum of 192 of them are bound via gold staples: approximately 87% compared to 91% found in the TRAP^K35C-Au^-cage (240 of 264).

The bowtie holes are positioned on the poles of the particle, and each is flanked by two four-fold holes resulting in some tension in the rings accommodated by differences in dihedral angles between the rings on the opposite sides of the holes (Figure S6). Significant differences in angles around four-fold holes and bowtie holes suggest that large flexibility of the constituent proteins allows necessary deformation. These deformations result in a decrease in the overall symmetry of the particle to C2 in comparison with TRAP^K35C-Au^ large^18^ and small^24^ cages (octahedral and tetrahedral, respectively). The angles formed also depend on the type of the ring. We define a ring type according to its surroundings. We can distinguish two major types of rings: type I rings that have four neighbors (Figure S7a) and type II rings with five neighbors (Figure S7b–e). Type II rings can be further divided into subgroups: IIa, IIb, IIc, and IId. All subgroups differ by spatial localization of holes and hole shapes (four-fold/bowtie). Types IIc and IId are symmetrically equivalent. Dihedral angles around Type I rings varies from 129.1° ± 0.6° to 134.5° ± 2.5°; in Type II rings, the differences change somewhat between subgroups and are as follows: type IIa – 129.1° ± 0.6° to 134.2° ± 1.7°, type IIb – 128.8° ± 1.0° to 134.4° ± 1.5°, type IIc – 129.5° ± 1.0° to 141.4° ± 0.2°, and type IId – 128.8° ± 1.2° to 138.6° ± 0.0° (mean ± SD).

Interestingly, the cryo-EM densities show some evidence of cargo, most likely TRAP rings in cages with rather low occupancy, suggesting an average of less than one such cargo ring per cage (Figure S4). In detail, we do not see any cargo inside the cage at map RMSD = 3.5, see limited evidence at RMSD = 2.5 and a ball-like shape, consistent with the dimensions of a TRAP-ring at RMSD = 1.5.

The mathematical model and previous work^18^ imply that ten cysteines on each ring are bound via Au(I) to partner cysteines on other rings. This would result in each cage having 96 Au(I). However, this is the upper limit, and given the relatively high distortion observed in the cage, we expect a somewhat smaller number. Indeed, as noted above, some bridging densities appear lacking in the maps. To confirm the presence of Au(I) in the cage structures and gain initial quantitative insight, we carried out electro thermal atomic absorption (ETAAS) analysis of the purified TRAP^S33C-Au^-cage (Table S2). This gave an average of 50 Au atoms per cage, representing a realistic lower limit.

### Geometrical Analysis and Mathematical Modeling

Eleven-fold rotational symmetry of the TRAP ring is not in compliance with the geometries of convex regular solids;^25^ however, we know that inherent protein flexibility may allow for small deformations from regularity to be included in mathematical modeling so that the final product is almost regular.^18^ Driven by this example, we were trying to build a predictive model for the TRAP^S33C-Au^-cage made out of 20 equivalent rings but failed. Cryo-EM three-dimensional (3D) reconstruction results revealed, however, that the TRAP^S33C^ cage indeed consists of 20 rings but those are split into five subgroups of 4 rings – one group comprises rings that have just four adjacent rings, the other four subgroups comprise rings having five adjacent rings but in different configurations with holes in the neighborhood (Figure S7). Knowing this exact topology of the assembly, we were able to model the cage mathematically. For this purpose, we harnessed the second part of the algorithm used in Malay et al.^18^ assuming an identical ring-ring connection type in the TRAP^S33C-Au^-cage as in the TRAP^K35C-Au^-cage. The outcome model (Figure 4b–d, bottom panels) matches the anticipated size and angles between pairs of rings (Table S3). Interestingly, its relative deviations from regularity for internal angles of a regular hendecagon (rda = 7.24%) and edge lengths (rdl = 7.64%) are substantially larger than that for the TRAP^K35C-Au^ model, where the rda and rdl values were 0.27 and 0.50%, respectively.^18^ This is a helpful guideline for future mathematical modeling and structure prediction as the models with similar levels of geometric distortions could be considered possible to be formed from the protein building blocks. We would like to stress that the mathematical models of protein cages can possess symmetries that are not exhibited by the actual cage because the structure and arrangement of the constituting proteins can break the theoretical symmetries that are derived from the homogeneous polyhedral cage scaffoldings constructed as part of the modeling. For example, the symmetry of the mathematical model from Figure 4 and the symmetry of the actual TRAP^S33C-Au^-cages are not the same. For the mathematical model, the underlying symmetry is S4 (combination of the C4 rotation and reflection by the plane perpendicular to the C4). Thus, because of the chiral nature of amino acids, none of the reflection operations are allowed. This leads us to decrease the roto-reflexion S4 point group to roto-inversion group 4̅ (combination of C4 rotation and inversion). Such symmetry operation does not affect the chirality of the amino acids. Cryo-EM analysis described above showed us that the overall symmetry is even lower and reduced to C2. This fact can be explained by extended flexibility around the poles of the protein cage and large differences in dihedral angles around opposing bowtie holes.

## Discussion

Artificial protein cages are free from restrictions imposed upon naturally occurring cages as a result of them having to be synthesized and assembled in cells. While some natural cages may have apparent advantages in their inherent ability to capture biological macromolecules, artificial protein cages can be designed de novo, in principle allowing capability of cargo capture to be incorporated in a bespoke manner. Overall, artificial protein cages have the potential to explore new structure and functional spaces not yet colonized by natural counterparts, unencumbered by evolutionary history. The first TRAP-cage (TRAP^K35C^-cage) addressed a number of these issues – using gold ion-links to join the protein building blocks of the cage together in place of the protein–protein interfaces found in natural complexes. It also widened the geometrical space available to protein cage designers – showing that an 11-sided (hendecagonal) protein (TRAP) could be used as a building block to build an apparently regular-faced convex polyhedron. This implies that further proteins with shapes previously thought unlikely to yield useable containers may in fact be viable cage building blocks. The current results were made possible at least in part by the fact that protein structures have sufficient inherent flexibility to absorb small “errors” required in such an assembly. Interestingly, the original TRAP-cage was constructed from 24 TRAP rings and likely represents the most stable possible cage that can be constructed from such a building block^23^ and certainly represents a structure in which the maximum number of available cysteines take part in a Cys–Au–Cys bond (11 out of 10 in each ring).

The question then arises if the TRAP^K35C^-cage was a rare, fortuitous event or if such proteins (even the same proteins) can be engineered, particularly given that other structures may be less energetically favorable and may require greater deformation of the constituent proteins likely leading to lower stability.

From a mathematical modeling point of view, expanding the search space to polyhedra bearing nonspherical symmetry point-groups allowed us to find theoretical solution that matches the experiment to some extent. This is very important because of amino-acid chirality. Asymmetric carbon disallows the use of any symmetry point groups that possess reflection planes because it would simply change l-amino acids into d-amino acids. We have to narrow the search space to rotation-based symmetry point groups. This can be simply achieved when we consider proper symmetry point groups only. The problem arises when we enter improper symmetry groups like S-groups. These groups are made out of rotation about an axis combined with reflection in a plane perpendicular to that axis. In this particular case, we faced the problem of how to preserve asymmetric carbon chirality, when the overall symmetry of the resulting polyhedral cage is indeed S-symmetrical. The only way to go around this problem is further symmetry decrease from roto-reflection to roto-inversion symmetry. Roto-inversion symmetry is a combination of rotation about an axis with the inversion center of a molecule. In that case, we can still operate on chiral amino acids without any limitation due to geometry and keep the spherical shape of the cages. Further decrease in symmetry leads to simple rotational symmetry point groups such as C2.

In this work, we confirm that alternative geometrical arrangements of TRAP rings in TRAP-cage structures, less energetically favored than the 24-ring cage, can be enforced by changing the position of the linker cysteine residue and thus the angle between the rings. This changes the most favored structure to a different geometrical arrangement of fewer (20) TRAP rings. While this structure is less stable than the original 24-ring cage, it nevertheless is able to form, presumably through larger deformation of the proteins. This is calculated to be 7.24 and 7.64% away from ideality for hendecagonal internal angles and edge lengths, respectively, compared to 0.27 and 0.50% in the original TRAP-cage, representing a further widening of geometrical space available to artificial protein cages while still retaining relatively high stability.

The new structure requires that each TRAP ring is connected to four or five ring neighbors, meaning that some rings have three nonbonded cysteines, rather than the single cysteine found in the original TRAP-cage. Given the usefulness of thiol groups in various connection chemistries, this may be suitable for future external decoration, bearing in mind that attachment of groups to the outside of artificial cage is of increasing interest.^1,26−29^

A small number of TRAP^S33C-Au^-cages may have auto-encapsulated a TRAP ring cargo (albeit with low frequency). This may be a simple stochastic process or, given that the engineered C33 residue is closer to the lumen compared to the original cysteine mutation (at position 35) and the fact that there is a greater number of cysteines not participating in the ring-ring cross-links, it may be possible that occasional disulfide bonds occur between a free cysteine on a TRAP ring in the cage wall and a free TRAP ring inside the cage. It should be noted that in earlier work,^18^ the ability to capture a TRAP ring inside TRAP cages has been observed in the case of cages built from TRAP^K35C^. Further investigation and optimization of this reaction could have potential for protein loading into TRAP-cages.

## Conclusions

In this work, we have shown that by changing the position of an introduced cysteine reside on the TRAP ring, we do not abolish its ability to form artificial cages but rather change the resulting cage geometry. This geometry is enforced through the different dihedral angles formed between adjacent rings. As a result, the previously constructed TRAP^K35C-Au^-cage consisting of 24 TRAP rings was converted to a new cage consisting of 20 TRAP rings.

**Figure 5:**
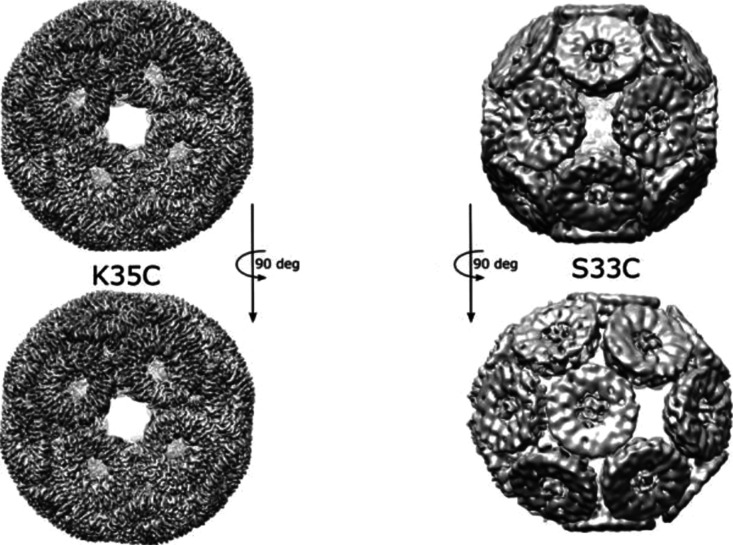
Comparison of TRAP^K35C-Au^- and TRAP^S33C-Au^ cages. (a) Two orthogonal views of the TRAP^K35C-Au^-cage (EMD-4443) and (b) two orthogonal views of the TRAP^S33C-Au^-cage showing decrease in the overall symmetry of the S33C-based cage in comparison with K35C-based one.

Additionally, from an evolutionary perspective, it is interesting to note how a small shift in the position of a single mutation (C35 to C33) results in a significant change in the cage size and geometry (Figure 5).

## Methods

### Cloning and Over-Expression

The gene-encoding S33C TRAP mutant variant was de novo chemically synthesized and cloned into appropriate plasmids. In a typical purification, *Escherichia coli* BL21(DE3) cells (Novagen) transformed with the pET21b(+) plasmid harboring the S33C TRAP gene, or pET151/D-TOPO for genes encoding TRAPs with alternative cysteines S33C and R64S, were grown at 37 °C with shaking in 3 L of LB medium with 100 μg mL^–1^ ampicillin until an optical density at 600 nm (OD 600) of 0.6 was reached, induced with 0.5 mM IPTG, and then further shaken for 4–5 h.

### Protein Expression, Purification, and Characterization

Cells induced to overexpress target protein were collected by centrifugation, and the pellet was stored at −80 °C until use. Cells were lysed by sonication at 4 °C in 50 mL of 50 mM Tris–HCl, pH 7.9 or 8.5, 0.05 M NaCl in the presence of proteinase inhibitors (Thermo Scientific) and in the presence or absence of 2 mM DTT, and lysates were centrifuged at 66,063 × *g* for 0.5 h at 4 °C. The supernatant fraction was heated at 70 °C for 10 min, cooled to 4 °C, and centrifuged again at 66,063 g for 0.5 h at 4 °C. The supernatant fraction was purified by ion-exchange chromatography on an ÄKTA purifier (GE Healthcare Life Sciences) using 4 × 5 mL HiTrap QFF columns with binding in 50 mM Tris–HCl, pH 7.9 or 8.5, 0.05 M NaCl, ±2 mM DTT buffer and eluting with a 0.05–1 M NaCl gradient. Fractions containing TRAP were pooled and concentrated using Amicon Ultra 10 kDa MWCO centrifugal filter units (Millipore), and the sample was subjected to size-exclusion chromatography (SEC) on a HiLoad 26/600 Superdex 200 pg column in 50 mM Tris–HCl, pH 7.9, 1 M NaCl +2 mM DTT (“cage buffer”) at room temperature. Protein concentrations were calculated using the absorption at 280 nm measured on a Nanodrop UV absorbance spectrophotometer. SEC experiments shown are representative of experiments repeated at least once, each giving similar results. Purity and homogeneity of protein was assessed by SDS-PAGE and native-PAGE.

### Gold Compounds

Chloro[diphenyl(3-sulfonatophenyl) phosphine]gold(I) sodium salt hydrate (Au-TPPMS) was procured from STREM Chemicals, UK, and reconstituted in water to 5 mM stock concentration or in 50 mM Tris–HCL, pH 7.9, 50 mM NaCl before use.

### Cage Assembly

Construction of the TRAP-cage was achieved by mixing purified S33C TRAP and Au-TPPMS in aqueous solution. The classic standard cage formation conditions comprises equimolar amounts of the S33C TRAP monomer and Au-TPPMS in cage buffer. The exact concentrations of reactants were tailored for each reaction but were typically as follows: 1 mM S33C TRAP (8.3 mg/mL) and 1 mM Au-TPPMS. Reactions were incubated for at least 4 to 5 days at room temperature. The formation of the TRAP-cage was analyzed using NS-TEM and native-PAGE analysis. Any precipitated material (aggregated protein, often present in samples after incubation) was removed by centrifugation at 12,045 × *g* for 5 min. The assembled TRAP^S33C-Au^-cage was purified by SEC on Superose 6 Increase 10/300 GL (GE Healthcare). Fractions containing the cage protein were pooled and concentrated using an Amicon Ultra 100 kDa MWCO centrifugal filter unit. Fractions containing the TRAP-cage were collected, and their purity was confirmed by native PAGE. The concentration of the purified cage was determined by measuring absorbance at 280 nm compared to the total protein concentration in the starting solution.

### Chemical Stability Tests

Reducing and denaturing agents used for cage-stability tests (DTT, TCEP, GSH, GSSG, SDS, and urea) were reformed in water or cage buffer, and the pH was adjusted when needed. Each sample with denaturing agents was concisely centrifuged in a desktop centrifuge, and a portion of supernatant was removed and mixed with 4× native PAGE sample buffer and subjected to native-PAGE analysis. Cage-stability experiments were repeated at least twice, each giving similar results.

### Thermal Stability Tests

Thermal stability of the purified TRAP^S33C-Au^-cage was assessed using a Tycho instrument (Nanotemper technologies). The system measures the fluorescence of intrinsic tryptophan and tyrosine residues detected at both 350 and 330 nm as a 30 °C/min temperature ramp is applied from 35 to 95 °C. Samples were measured using standard settings.

### pH Stability Tests

For testing the effect of pH on cage assembly, Au-TPPMS stocks were prepared in 50 mM sodium acetate at pH 4.0, 50 mM potassium phosphate at pH 6.0 and 12.0, 50 mM Tris–HCl at pH 8.0 and 50 mM glycine–NaOH at pH 10.0, 50 mM glycine–HCl pH 2.0 and 3.0, and 50 mM potassium chloride pH 12.0.

### Transmission Electron Microscopy

Samples were typically diluted to a final protein concentration of 0.035 mg ml^–1^, centrifuged briefly in a desktop centrifuge, and the supernatant was applied onto hydrophilized glow-discharged carbon-coated copper grids, negatively stained with 3% phosphotungstic acid, pH 8, and visualized using a JEOL JEM-1230 80 kV instrument. All TEM results shown were repeated at least twice, independently, each giving similar results.

### Native PAGE

Samples were run on 3–12% native Bis-Tris gels, following the manufacturer’s recommendations (Life Technologies). Samples were mixed with 4x native PAGE sample buffer (200 mM BisTris, pH 7.2, 40% w/v glycerol, 0.015% w/v bromophenol blue). As a qualitative guide to the molecular weights of migrated bands, NativeMark unstained protein standard (Life Technologies) was used. Where blue native PAGE was performed, protein bands were visualized according to the manufacturer’s protocol (Life Technologies), otherwise InstantBlue protein stain (Expedeon) was used. All native PAGE gels were repeated independently at least twice with each repeat giving similar results.

### Cryo-EM Single-Particle Reconstruction of TRAP-cage Formed Using Au-TPPMS

Cryo-EM single-particle reconstruction of TRAP-cage was formed using Au-TPPMS. A purified sample (20 μL of 2.3 mg/mL) formed using Au-TPPMS was applied to glow-discharged holey carbon grids (Quantifoil R 1.2/1.3, Mo 200 mesh) with a thin amorphous carbon film of around 10 nm thickness over the holes and incubated for 30 s at 4 °C and 100% humidity. Grids were then blotted for 3.0 s and plunged into liquid ethane using a Vitrobot Mark IV (FEI). Data were recorded semi-automatically using the EPU software on a transmission electron cryo microscope (FEI Titan Krios) operated at an accelerating voltage of 300 kV and at a nominal magnification of 75,000×.

The CryoEM map was reconstructed using cryoSPARC v2.15.0,^30^ and the full pipeline is described in Figure S3.

All figures displaying cryoEM maps and/or pseudo atomic models were prepared in USCF Chimera^31^ or ChimeraX.^32^

### Mass Photometry

Borosilicate coverslips (#1.5, 24 × 50 mm^2^, THORLABS) were cleaned by sequentially submerging and sonicating for 5 min in water, isopropanol, and water again, before being dried under a clean stream of nitrogen. All movies were obtained on a OneMP (Refeyn Ltd., Oxford, UK) using a frame-binning of 5 and a pixel-binning of 4, and recorded for 100,000 frames (∼17 mins) with an acquisition speed of 100 frames per second. Native movies were then processed with DiscoverMP (v2.0.3, Refeyn Ltd., Oxford, UK) using a further averaging frame size of 5 and with reflectivity correction. All other parameters were set to the default.

To monitor the dissociation induced by reduction, clean gaskets (Grace Bio-Labs reusable CultureWell) were placed on a clean coverslip with 9 μL of buffer (50 mM TRIS, 50 mM NaCl) added to the well. The coverslip was positioned in the optimal focus position as determined by the sharpness of the glass image. The TRAP^S33C-Au^-cage (1 μL) at a concentration of 126 μg/mL was added to the well for a working concentration of approximately 12.6 μg/mL, and recording started shortly after. Approximately 3 min in ∼frame 18,000, 1 μL of 20 mM DTT stock was diluted into the sample during acquisition to achieve a working concentration of 1.8 mM in order to promote dissociation of the TRAP complex.

### High-Speed Atomic Force Microscopy

Prior to the HS-AFM experiment, TRAP^S33C-Au^-cages were diluted to 17 μg/mL with a 50 mM Tris pH 7.9 and 50 mM NaCl. The sample (2–3 μL) was applied to freshly cleaved mica. HS-AFM^33,34^ (SS-NEX, RIBM, Japan) with a laboratory-built phase shift amplitude detector^35^ and an automated force controller^36^ was used. Ultra-Short Cantilevers (USC-F1.2-k0.15, NanoWorld, Switzerland), with a nominal spring constant of 0.15 N/m were excited at 550–650 kHz with a free amplitude of 2–3 nm (peak to peak). Images were taken at 1 frames/s with 200 × 200 pixel at 200 × 200 nm or 80 × 80 nm. The final concentration of DTT (1 mM) was added to the observation buffer by pipetting during HS-AFM observation. The obtained sequential HS-AFM images were analyzed and processed using laboratory-made IgorPro (WaveMetrics, USA)-based functions. First, the frames were contrast-adjusted, then lateral drift was corrected using an registration algorithm in ImageJ. The processed image was constructed with time-averaged over 10 frames to enhance noise signal ratio, and high-pass FFT filter was used to identify the TRAP rings structure on the TRAP cage surface.

### Electrothermal Atomic Absorption Spectrometry

The sample (2 mg) was dissolved in 25 mL of 0.2% HCl. The solution was diluted 25 times, and the total gold determination was performed using an electrothermal atomic absorption spectrometer (PinAAcle 900Z, Perkin Elmer), with Zeeman background correction, at a wavelength of 242.80 nm (slit width of 0.7 nm). The measured volume of the sample solution was 10 μL, and a mixture of matrix modifiers (5 μg of Pd(NO_3_)_2_ and 3 μg of Mg(NO_3_)_2_) was added to each sample. Three sets of measurements were carried out for each sample, with each set consisting of three repeats.

### Mathematical Modeling of the S33C-TRAP-cage Geometry

The TRAP rings were modeled as regular hendecagons (11-sided polygons), and all the bonds in-between the rings were encoded as edge-to-edge gluings in-between the hendecagons in the file describing the topology of the cage (i.e., all the links between the hendecagons) that was fed into the following optimization program.

The C++ program (of around 8400 lines of code) was harnessed to obtain the geometric attributes of the cage. First, the hendecagonal faces were modeled as rigid bodies, with adjacent faces linked by two Hookean springs with a rest position set to 5% of the edge length. The energy of the system was then minimized via Monte Carlo algorithm. The coordinates of the resulting assembly were then used by the program to model the cage as a set of rigid rods using an energy functional divided into three terms: edge length deviations, face edge angular deviations, and the degree of nonplanarity. The first one measured by how much the edge lengths deviated from a chosen reference length. The second one quantified by how much the angle between the hendecagon edges deviated from the internal angle of the regular hendecagon. The third term computed the level of nonplanarity of the hendecagons. Each term was assigned a weight factor, with the planarity weight set to three orders of magnitude larger than for the lengths and the angles. This gave us structures with preserved planarity (with zero planarity distortions modulo numerical error). The energy functional was minimized using a Monte Carlo method and the program output a file containing the vertex coordinates, the topology of the cage, as well as the order of deformations obtained for the angles and edge lengths. The exact explanation of the algorithm can be found in a previous study,^23^ but some details were also covered previously.^37^ From the optimized geometries generated using the method described above, we selected one candidate for which the dihedral angles and diameters were in line with the cryo-EM measurements, and the relative deformation levels for edge lengths (rdl) and angles (rda) were less than 10%. Rdl is defined as the largest absolute value of the difference between the edge lengths and the average edge length, divided by the average value between all the faces. Rda is defined similarly for the angles.

## Supporting Information Available

Mass photometry; pH and thermal stability; procedure for cryo-EM reconstruction of TRAP^S33C-Au^-cage; cryo-EM resolution data; dihedral angles of adjacent rings across the four-fold and bowtie holes in the cage; cryo-EM maps highlighting missing densities for Au bridges; dihedral angles between adjacent rings; table of cryo-EM data collection and refinement statistics; table of results of ETAAS analysis of TRAP^S33C^-^Au^-cage for gold content; table showing comparison of dihedral angles between experimental and theoretical models of TRAP^S33C-Au^-cage; and HS-AFM movies of TRAP^S33C-Au^-cages (PDF)
High-speed atomic force microscopy showing the intact TRAP^S33C-Au^-cage (MP4)
High-speed atomic force microscopy showing the effect of 1 mM DTT addition to the TRAP^S33C-Au^-cage (MP4)
