# Supplementary material for: Shape-Morphing of an Artificial Protein Cage with Unusual Geometry Induced by a Single Amino Acid Change
Source: ACS Nanosci Au. 2022 May 9;2(5):404–13. doi: 10.1021/acsnanoscienceau.2c00019 (PMC9585630; doi:10.1021/acsnanoscienceau.2c00019)
Supplement: Supplementary file 1 — ng2c00019_si_001.pdf [file ng2c00019_si_001.pdf]

Supplementary Information

Shape-morphing of an artificial protein cage with unusual geometry induced by a single amino acid change

Mohit Sharma[a,b], Artur P. Biela[a], Agnieszka Kowalczyk[a,c], Kinga Borzęcka-Solarz[a], Bernard M.A.G. Piette[d], Szymon Gaweł[a], Joshua Bishop[e, f], Philipp Kukura[e, f], Justin L. P. Benesch[e, f], Motonori Imamura[g, h], Simon Scheuring[g, h], Jonathan G. Heddle[a*]

a - Malopolska Center of Biotechnology, Jagiellonian University, Gronostajowa 7A, Kraków, 30-387, Poland

b – School of Molecular Medicine, Medical University of Warsaw, Warsaw, 02-091, Poland

c – Faculty of Mathematics and Computer Science, Jagiellonian University, Kraków, 30-348, Poland

d – Department of Mathematical Sciences, Durham, DH1 3LE, UK

e – Department of Chemistry, University of Oxford, Oxford, OX1 3TA, UK

f - Kavli Institute for Nanoscience Discovery, University of Oxford, Oxford, OX1 3QU, UK

g - Department of Anesthesiology, Weill Cornell Medicine, New York City, NY 10065, USA.

h – Department of Physiology and Biophysics, Weill Cornell Medicine, New York City, NY 10065, USA
* – Correspondance email: Jonathan.heddle@uj.edu.pl
Mohit Sharma and Artur P. Biela contributed equally to this work

Supplementary Figures

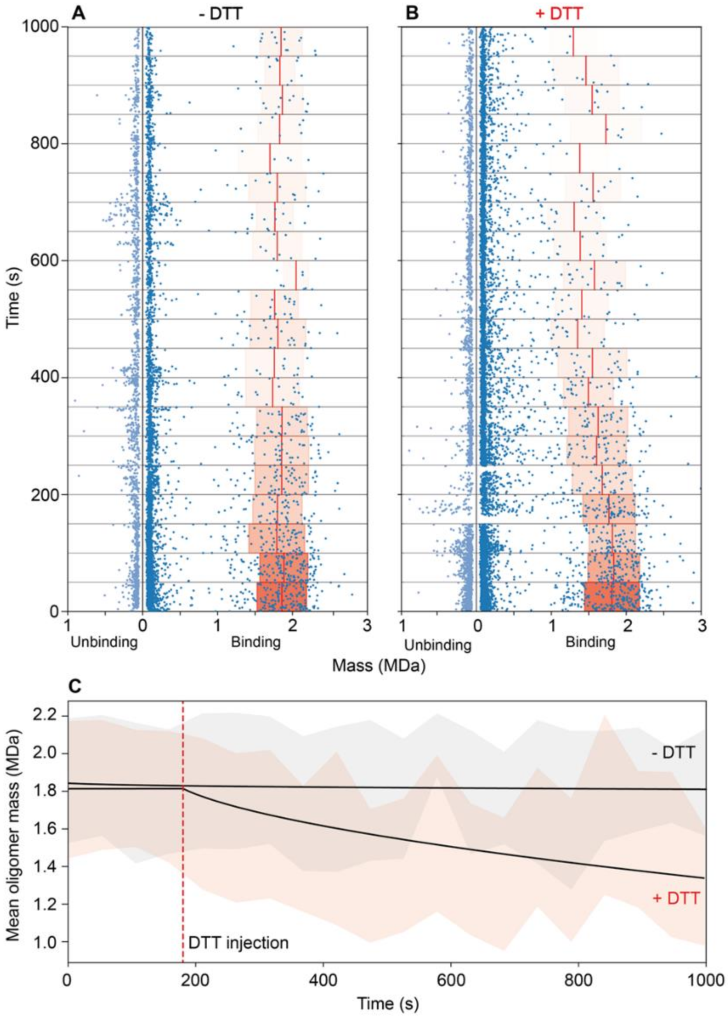

Figure S1: Mass photometry of TRAP$^{S33C\text{-}Au}$-cage. Mass photometry data for TRAP$^{S33C\text{-}Au}$-cages without (**A**) and with (**B**) DTT. Events corresponding to molecular cages were detected over 1000 s of acquisition. Events corresponding to the binding of intact cages were binned into blocks of 50 s and the distribution of events over 900 kDa was calculated. Distributions are represented by a thick red line at the mean with a red band extending to ±1 standard deviation. The opacity of each block is scaled linearly according to the number of events therein. **C)** Corresponding distributions of **A** and **B** showing the average dissociation of TRAP cages over time following an injection of DTT. Trends were fitted to an exponential decay from the moment of injection**.**

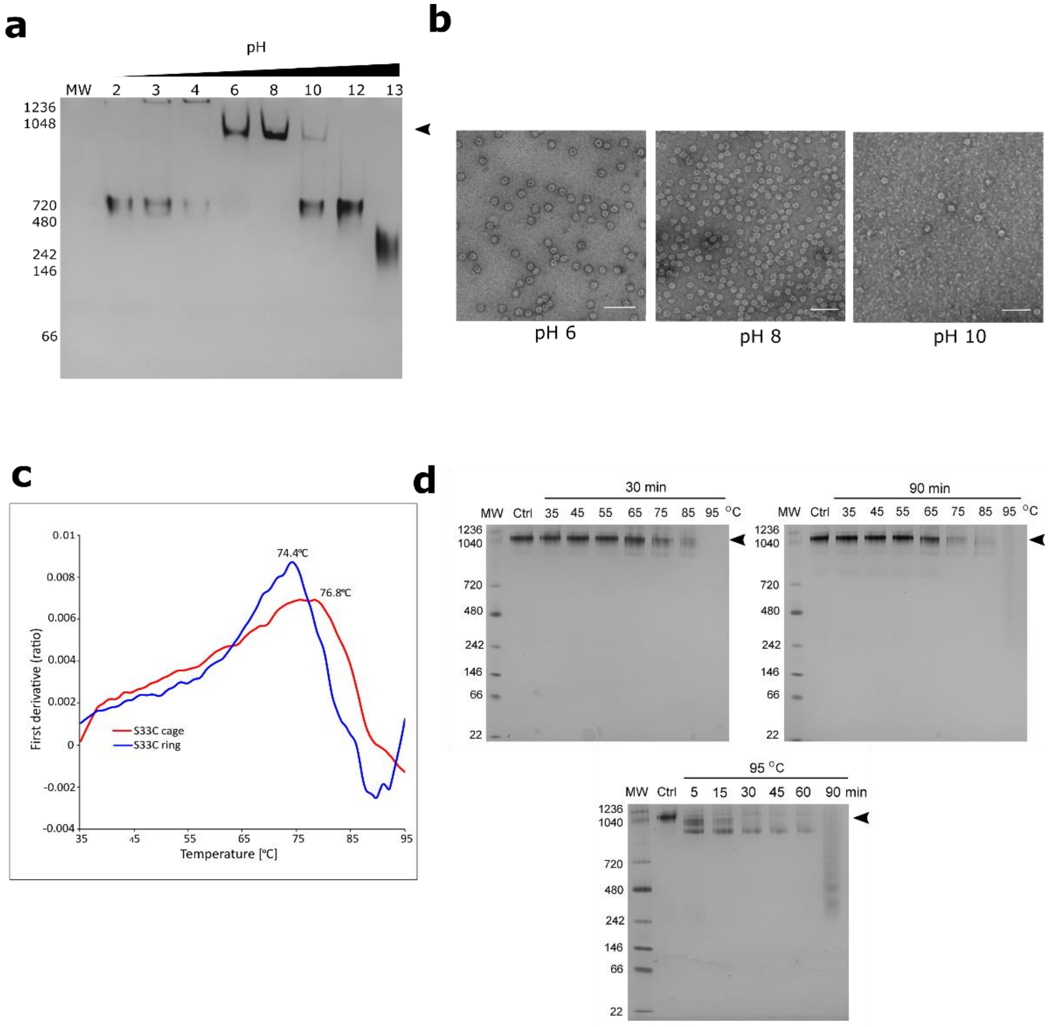

Figure S2. pH and thermal stability of TRAP$^{S33C-Au}$-cage. a, Native-PAGE gel showing that effect of pH on TRAP$^{S33C-Au}$-cage stability. **b,** TEM micrographs of TRAP$^{S33C-Au}$-cage at indicated pH values. **c,** thermal denaturation curves with indicated inflection temperature showing that TRAP$^{S33C-Au}$-cage is slightly more stable than isolated rings; T_i^{ring} = 74.4 ^{o}C and T_i^{cage} = 76.8 ^{o}C **d,** native-PAGE gel showing thermal stability after 30 (top left) and 90 (top right) minutes incubation at different temperatures, together with time course at 95 °C (bottom panel). Black arrowheads on gels indicate position of band corresponding to TRAP$^{S33C-Au}$-cage.

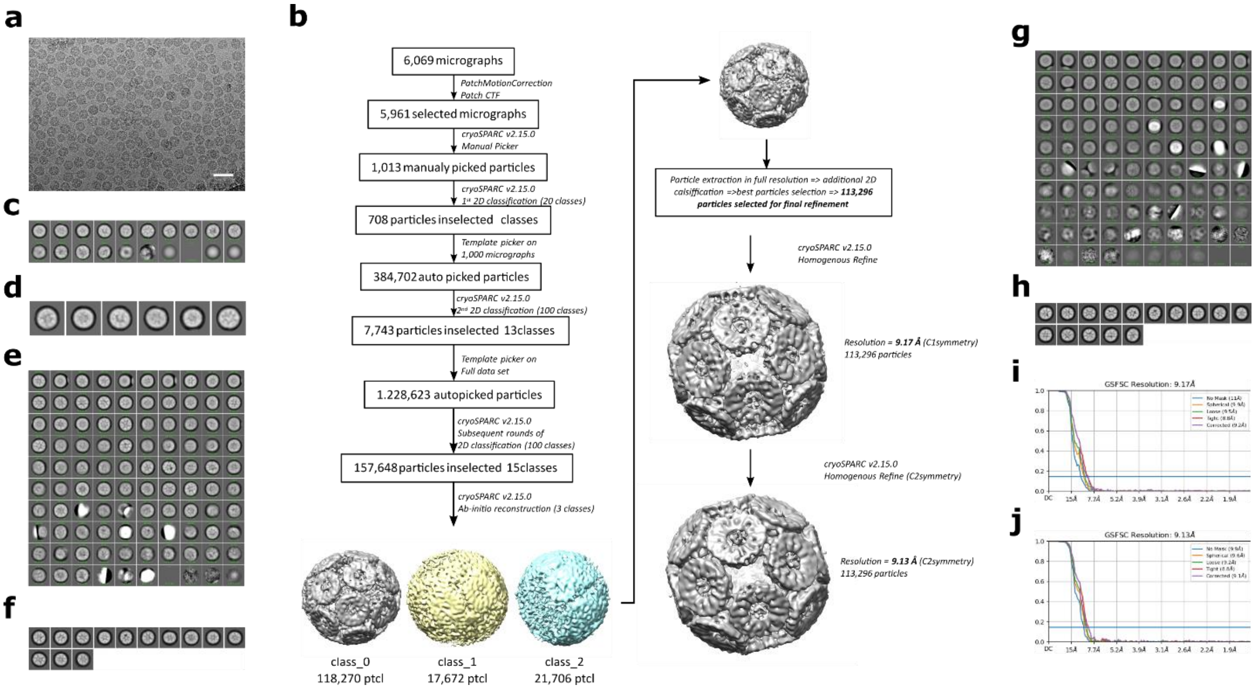

Figure S3. Procedure for cryo-EM reconstruction of TRAP$^{S33C\text{-}Au}$-cage. a, representative micrograph. Scale bar – 50 nm. **b,** Summary of the image processing procedure (see Methods). **c,** 2D class averages from first reference-free 2D calssification in cryoSPARC **d,** selected 2D class averages from reference-free 2D calssification used for first automated template picking on 1,000 micrographs **e,** initial 2D class averages after template picking. **f,** final 2D class averages used for template picking on full data set. **g,** initial reference free 2D classification on full data set. **h,** final selected 2D class averages. **i,** gold standard FSC correlation curve for C1 refined structure. **j,** golden standard FSC correlation curve for C2 refined structure.

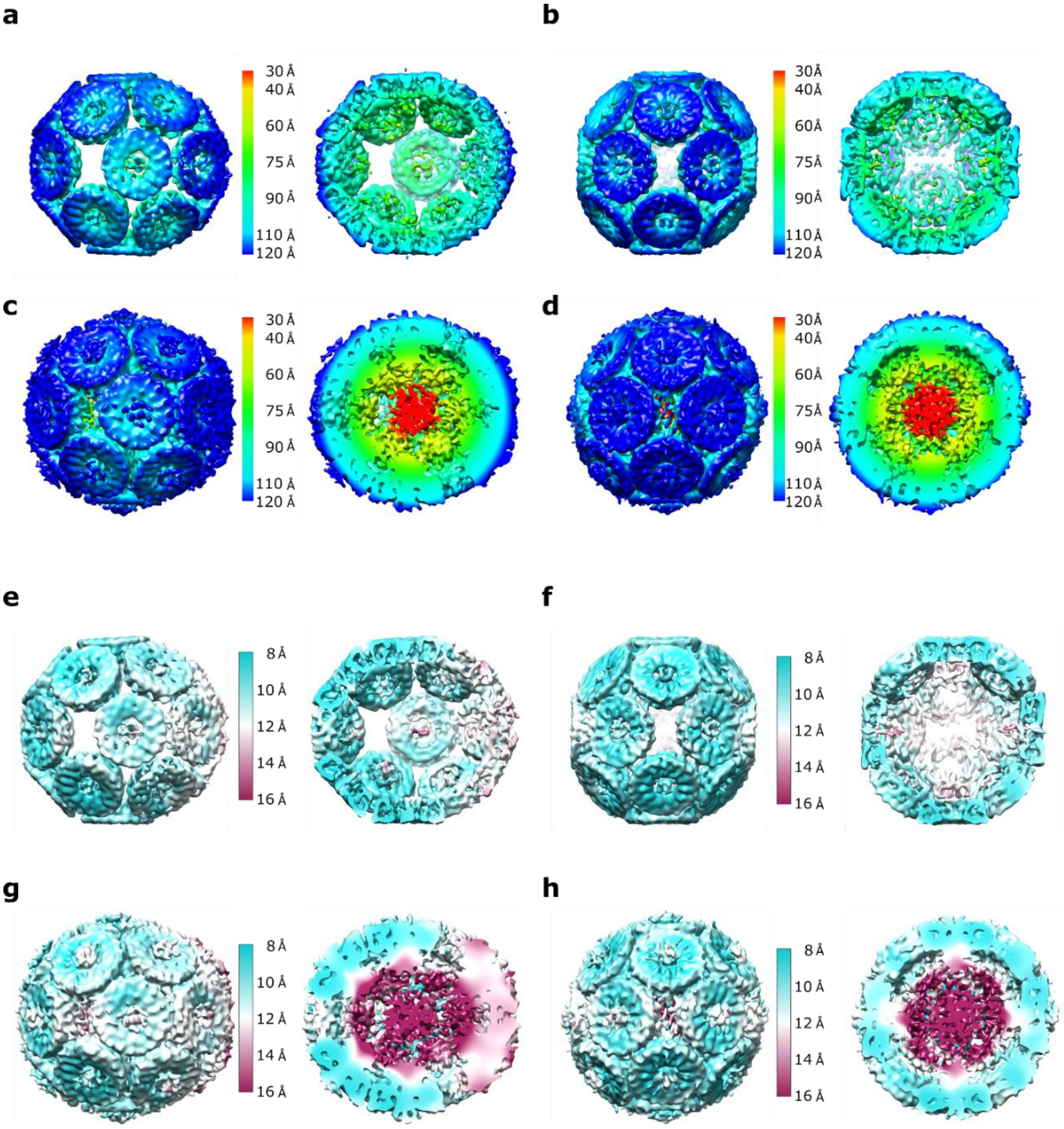

Figure S4. Radius dependent and local resolution dependent coloring of TRAP$^{S33C\text{-}Au}$-cage. Two orthogonal views (**a** and **b, c** and **d**) coloured accordingly to distance form the cage center. Panels, **a** and **b** are contoured at RMS level 3.5, while panels **c** and **d** are contoured at RMS level 1.5 to highlight presence of the cargo and slight cage deformation from the ideal sphere. Two orthogonal views (**e** and **f, g** and **h**) coloured accordingly to local resolution. Panels, **e** and **f** are contoured at RMS level 3.5, while panels **g** and **h** are contoured at RMS level 1.5 to higlight presence of the cargo and its random orientation in the cage lumen. Right panels are cross sections of the left panels.

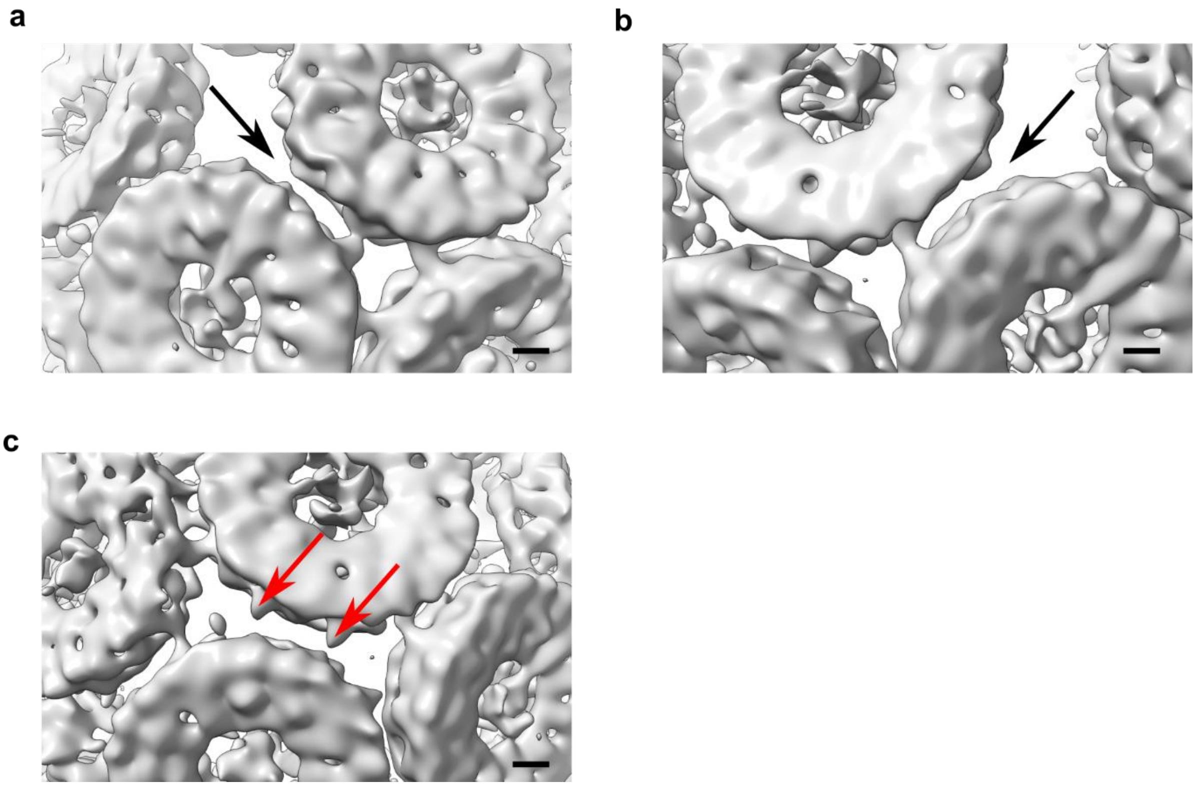

Figure S5: Missing densities for Au bridges. a,b black arrows showing lack of density of one bridge from the pair; **c** red arrows showing incomplete densities for both Au bridges, indicating partial occupancy at the indicated positions. Maps contoured at RMDS = 3.5; scalebar – 10 Å

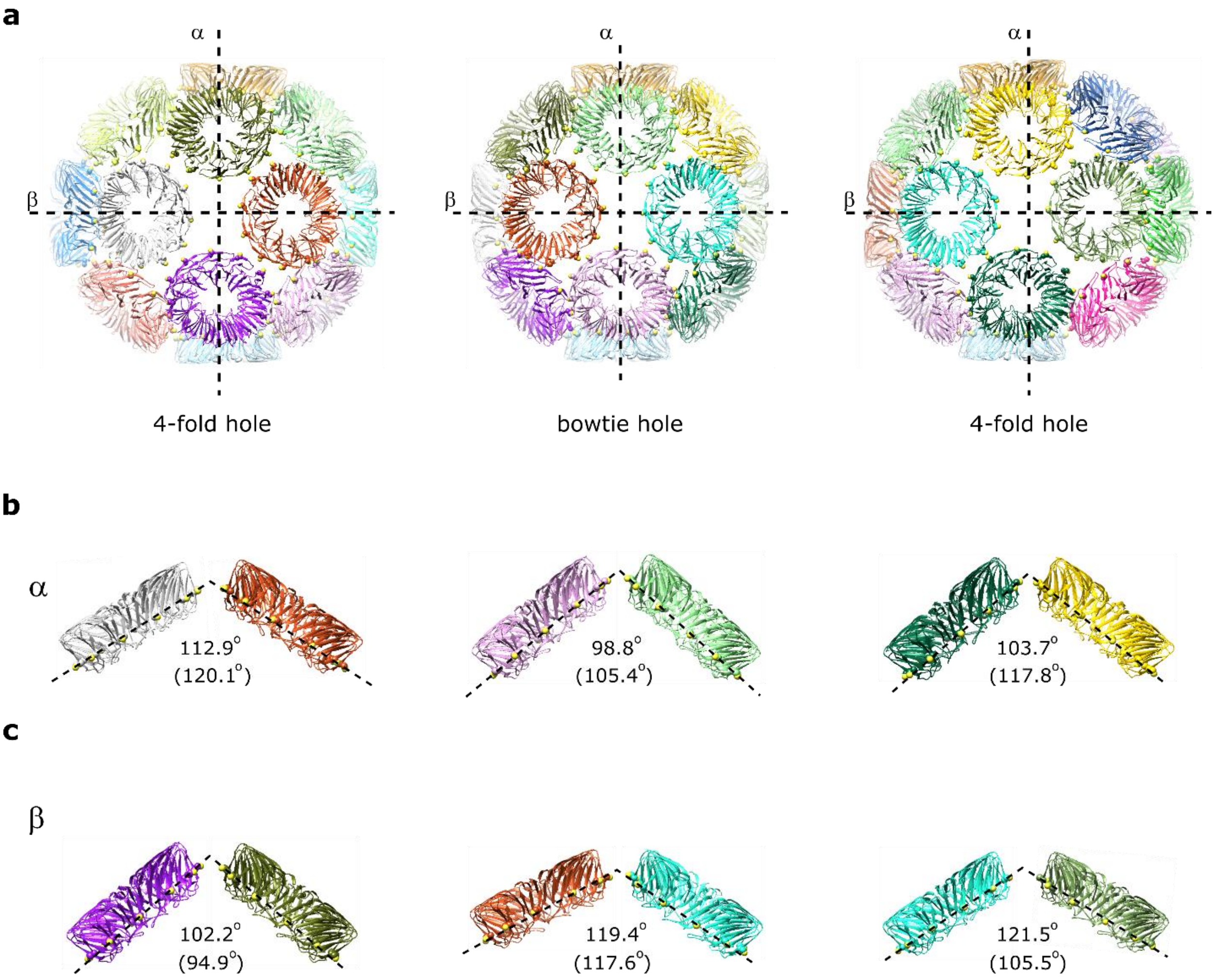

Figure S6. Dihedral angles of adjacent rings across the 4-fold and bowtie holes. a, definition of angles **b,** measures of α angles **c,** measures of β angles; values in brackets are measure of symetrically related angles

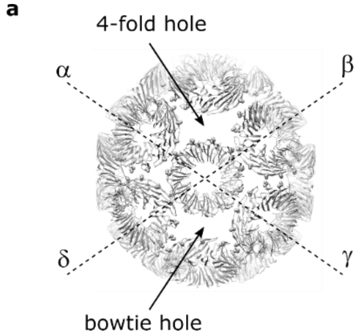

#	angle [°]	SD [°]
α	130.3	2.6
β	130.3	0.1
γ	134.5	2.5
δ	129.1	0.6

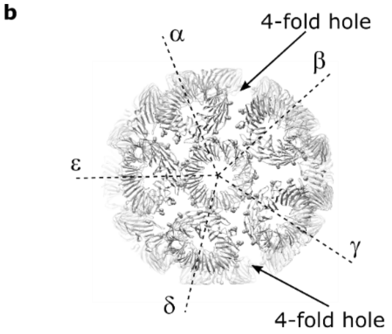

#	angle [°]	SD [°]
α	131.0	1.1
β	129.1	0.6
γ	131.5	2.5
δ	131.5	0.4
ε	134.2	1.7

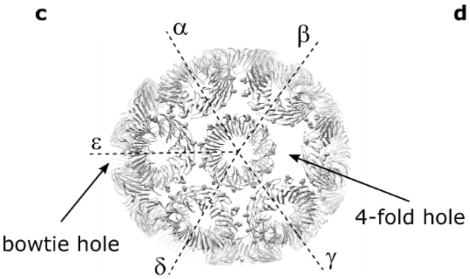

#	angle [°]	SD [°]
α	129.5	1.0
β	134.4	1.5
γ	133.9	0.3
δ	128.8	1.2
ε	134.2	0.2

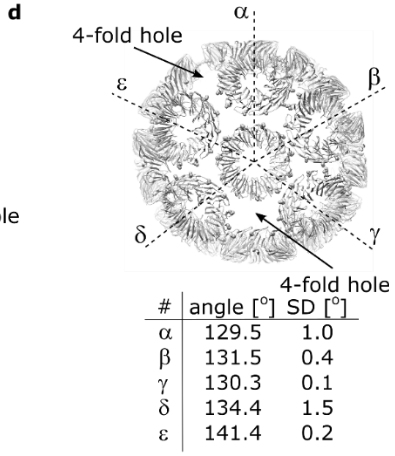

#	angle [°]	SD [°]
α	129.5	1.0
β	131.5	0.4
γ	130.3	0.1
δ	134.4	1.5
ε	141.4	0.2

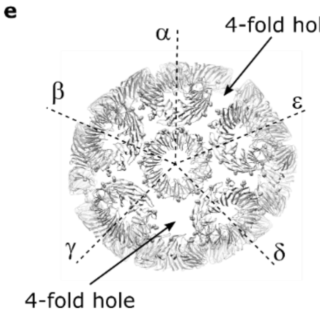

#	angle [°]	SD [°]
α	128.8	1.2
β	131.0	1.1
γ	130.3	2.6
δ	133.9	0.3
ε	138.6	0.0

Figure S7: Dihedral angles between adjacent rings. a, Type I rings (4 in total, each flanked by one 4-fold hole and bowtie hole) **b-e,** Type II rings (16 in total, each with 5 neighbours **b,** Type IIa **c,** Type IIb **d,** Type IIc **e,** Type IId. On each panel, an arrow indicates a hole to facilitate rings localization. Rings of Type IIc and IId are symetrically equivalent. Greek alphabet letters indicate specific angles.

Supplementary Tables:

Table S1: Cryo-EM data collection and refinement statistics

**Data collection and processing**	*TRAP$^{S33C\text{-}Au}$-cage*
Microscope	FEI Titan Krios
Detector	Gatan K3
Magnification	105,000
Voltage (kV)	300
Defocus range (µm)	-0.9 ~ -3.4
Pixel size (Å)	0.86
Total electron dose (e-/Å^2)	40
Number of frames per image	40
Number of images	6,069
Initial particle number	1.228,623
Symmetry imposed	C2
Final particle number	113,296
Map resolution (Å)	9.13
FSC threshold	0.143

Table S2: Results of ETAAS analysis of TRAP$^{S33C\text{-}Au}$ cage for gold content

	**mg(Au)/g(protein)**	**SD mg(Au)/g(protein)**	**Au/cage**	**SD (Au/cage)**
**sample**	5.14	0.2	48	2
**repeat 1**	5.28	0.1	49	1
**repeat 2**	5.56	0.1	52	1
**Avg**	5.33		50	

Table S3: Comparison of dihedral angles between experimental and theoretical model of TRAP$^{S33C\text{-}Au}$-cage. Faces numbering according to Supplementary Figure 7; 4 => Type I, 5A => Type IIa, 5B => Type IIb, 5C => Type IIc, 5D => Type IId

dihedral angles between faces	measured angles α_i between the rings	Dihedral angles β_i in theoretical model
angle 4-5C	130.3	131.20
angle 4-5D	130.3	131.14
angle 4-5A	129.1	129.37
angle 4-5A	131.5	129.42
angle 5C-5B	134.4	131.60
angle 5C-5C	141.4	140.05
angle 5C-5B	129.5	130.97
angle 5C-5A	131.5	131.59
angle 5A-5B	134.2	138.47
angle 5A-5D	131,0	131.58
angle 5A-4	129.1	129.40
angle 5B-5C	134.4	131.65
angle 5B-5D	133.9	131.64
angle 5B-5D	128.8	130.97
angle 5D-5D	138.6	140.06
angle 5D-5B	133.9	131.64
angle 5D-4	130.3	131.18
angle 5D-4	130.3	131.18
angle 5D-5A	131,0	131.58
angle 5D-5B	128.8	130.96
angle 5B-5A	134.2	138.45
angle 5B-5C	129.5	130.95
angle 5B-5C	134.4	131.64
angle 5A-4	129.1	129.41
angle 5A-4	131.5	129.37
angle 5A-5C	131.5	131.56
angle 5C-4	130.3	131.15
angle 5C-5B	134.4	131.66

angle 5C-5C	141.4	140.06
angle 4-5A	129.1	129.44
angle 4-5D	130.3	131.20
angle 5A-4	131.5	129.38
angle 5A-5C	131.5	131.54
angle 5A-5B	134.2	138.46
angle 5A-5D	131	131.61
angle 5A-5C	130.3	131.17
angle 5C-5B	129.5	130.93
angle 5B-5D	133.9	131.67
angle 5B-5D	128.8	130.98
angle 5D-5D	138.6	140.07
angle 5D-5B	133.9	131.63
angle 5B-5A	131	131.55
angle 5B-5B	128.8	130.95
angle 5B-5A	134.2	138.45
angle 5B-5C	129.5	130.96
angle 5A-4	131.5	129.40
angle 5A-5C	131.5	131.59
angle 5C-4	130.3	131.17
Total deviation	n/a	**14.88%**
edge deviation in the cage	n/a	**7.64%**
angle deviation in the cage	n/a	**7.24%**
min angle	128.8	**129.37**
max angle	141.4	**140.07**
mean angle	132.04	132.33
median angle	131.25	131.37
$\Sigma(\alpha_i-\beta_i)^2$	n/a	184.075

Movie S1.

High-speed atomic force microscopy (HS-AFM) movie frames, taken at 1 frame per second, 80 nm x 80 nm, 200 pixel x 200 pixel, showing the intact TRAP$^{S33C-Au}$-cage. Movie on the left is the original movie and movie on the right is processed with averaged 10 frame and high-pass FFT. Scale bar at *t* = 0 s indicates 20 nm. Z colour scale is set to 0 nm to 20 nm. This movie plays at 10 times of the original speed.

Movie S2.

High-speed atomic force microscopy (HS-AFM) movie frames, taken at 1 frames per second, 200 nm x 200 nm, 200 pixel x 200 pixel, showing the effect of 1 mM DTT addition to TRAP$^{S33C-Au}$-cage. 1 mM DTT (final concentration) was added during HS-AFM observation at *t* = 0s. Scale bar at *t* = 0s indicates 50 nm. Z colour scale is set to 0 nm to 20 nm. This movie plays at 10 times of the original speed.
